# Efficient communication dynamics on macro-connectome, and the propagation speed

**DOI:** 10.1038/s41598-018-20591-y

**Published:** 2018-02-06

**Authors:** Masanori Shimono, Naomichi Hatano

**Affiliations:** 1 Graduate School of Medicine and Faculty of Medicine, Kyoto University, 53 Kawaramachi, Shogoin, Sakyo-ku, Kyoto, 606-8507 Japan; 2 Riken Brain Science Institute, 2-1 Hirosawa, Wako, Satama 351-0198 Japan; 3 Institute of Industrial Science, The University of Tokyo, Komaba 4-6-1, Meguro, Tokyo, 153-8505 Japan

## Abstract

Global communication dynamics in the brain can be captured using fMRI, MEG, or electrocorticography (ECoG), and the global slow dynamics often represent anatomical constraints. Complementary single-/multi-unit recordings have described local fast temporal dynamics. However, global fast temporal dynamics remain incompletely understood with considering of anatomical constraints. Therefore, we compared temporal aspects of cross-area propagations of single-unit recordings and ECoG, and investigated their anatomical bases. First, we demonstrated how both evoked and spontaneous ECoGs can accurately predict latencies of single-unit recordings. Next, we estimated the propagation velocity (1.0–1.5 m/s) from brain-wide data and found that it was fairly stable among different conscious levels. We also found that the shortest paths in anatomical topology strongly predicted the latencies. Finally, we demonstrated that *Communicability*, a novel graph-theoretic measure, is able to quantify that more than 90% of paths should use shortest paths and the remaining are non-shortest walks. These results revealed that macro-connectome is efficiently wired for detailed communication dynamics in the brain.

## Introduction

The brain can be thought of as both a biological and a physical system, in which electrical signals propagate along axonal or dendritic wiring. The propagation pattern eventually emerges as various cognitive functions and internal thoughts. Propagations along underlining connectivity or wiring can be ubiquitously observed in biological networks^1^, the spread of infections^2^, and the organization of the internet^3^. To understand such propagation phenomena, quantitative evaluations that consider the constraints caused by underlying structural networks are critically important. Quantitative evaluations and interpretations have been supported by graph-theory-based approaches^4,5^. For instance, the comprehensive network (connectomics) approach is essential for studying brain wiring^6^, and graph-theoretic analyses have been used to study a range of relevant topics, such as the Small-World property, which can explain why spatially distant brain regions are able to communicate easily^7^, hubs and rich club organization, which can be used to extract a collection of highly-connected nodes^8^, and community architecture, which can characterize global groups of nodes^9^. The basic concepts of these approaches to network analysis have been previously summarized in textbooks on graph theory^10,11^.

Preceding graph-theoretic evaluations of detailed topologies, the extent to which structural networks are similar to functional or effective networks^12^, which can be reconstructed from recordings of long-term neuronal activity, is a fundamental question^13^. This issue is also essential for studying microscopic neuronal networks^14–17^. Recently, connectomics studies have been made possible due to the massive efforts of collaborating teams, and the quality and resolution of data have gradually improved^18–20^. The main focus of these studies is often structural networks or spatial patterns of relatively stable neuronal activities^21^. While characterizing relatively stable architecture, studies have gradually emphasized the importance of the dynamics of functional network architectures^22–24^. However, very few studies have satisfied the following criteria: (1) millisecond temporal resolution, (2) treating the whole-brain as one system, (3) inclusion of structural constraints, and (4) exclusion of computational demands of electrical current source estimates like E/MEG.

To address these criteria, we gathered multiple data sets recorded via three modalities: The first modality, ECoG, is a promising technique for capturing the propagation of electrical signals in a large cortical region^25^ or whole cortex^26^. We expected that combining ECoG data with neuronal spike signals would provide a neuronal or microscopic scheme of macroscopic brain signals^27^. As mentioned, we also included structural network data to express the anatomical fiber pathways^28–30^.

When theoretically testing electrical propagations along a set of pathways, it is possible to simply consider the contributions of the shortest paths. However, a recent study demonstrated the importance of considering the non-shortest paths^18^. A graph-theoretic measure, termed Communicability, provides a systematic framework for assessing the relative contributions of shortest and non-shortest paths (walks)^31,32^. However, Communicability has not yet been used to test the relationships among different neural propagations, which can spread throughout the whole brain within milliseconds.

In the present study, we asked four basic questions regarding the time taken for electrical signals to propagate through the brain: First, focusing on propagations of evoked electrical signals in the primate cortex, we asked how well the global transmissions of electrical signals recorded with ECoG could predict the onset timings of neuronal spikes. Second, to check the robustness of this predictive ability, we evaluated how well time delays in ECoG data could predict time delays in spontaneous neuronal spikes in the absence of a clear stimulus onset. Third, as a simple but fundamental question, we estimated the propagation velocity of globally propagating electrical signals. Fourth, we examined the possibility of creating fundamental graph-theoretic descriptions of propagation using Communicability.

## Results

### Comparison of evoked activities between ECoG and neuronal spikes

In past studies, the time delays necessary for information processes in visual pass way were known^33^.

**Figure 1:**
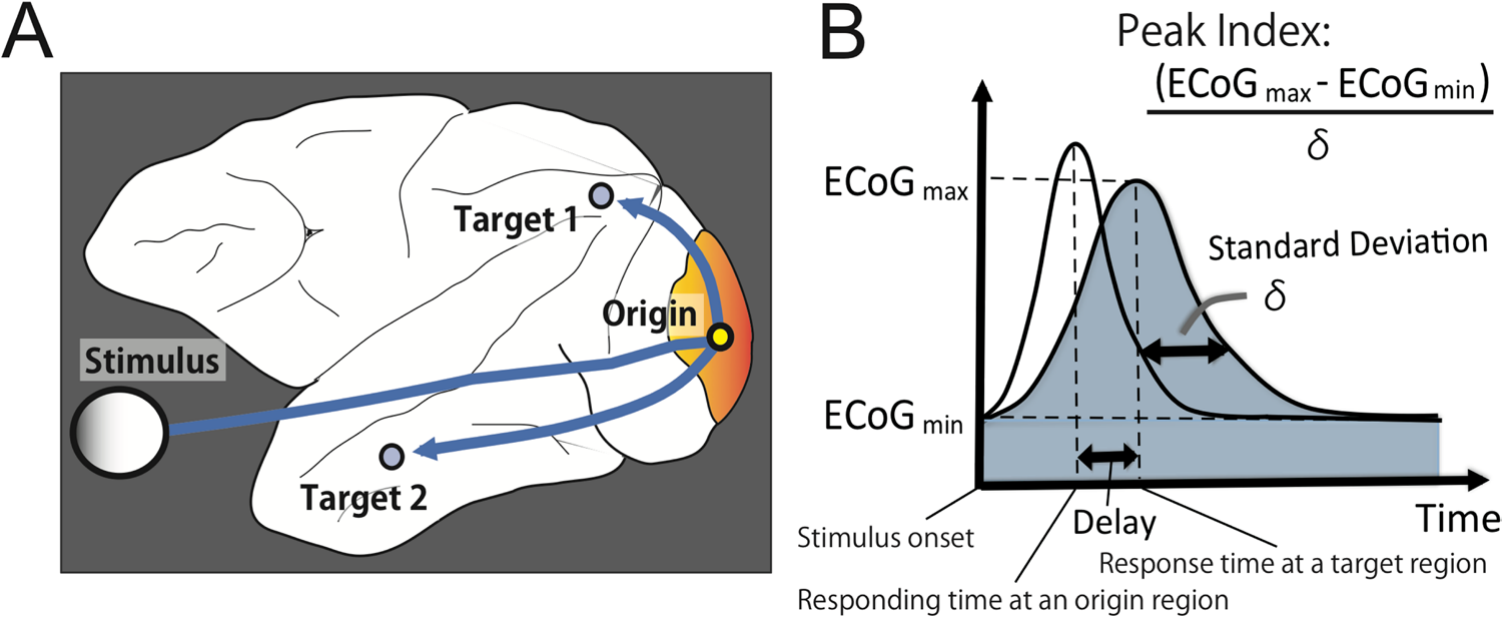
The definition of transmission delays in evoked experiments. (**A**) Schematic illustration showing prominent visually-evoked electrical activity transmitted via an “origin” brain region, such as the primary visual area (V1), to other “target” brain regions. (**B**) The transmission delays were simply determined as gaps of latencies of primary peaks of evoked activities between different brain regions. To evaluate the sharpness of evoked responses, we defined a variable named *Peak Index* using the equation shown in panel B. Finally, we used the averaged *Peak Index* for all “target” regions to optimize the size of the time window in which we searched for peak points of evoked activities.

As a double check of past studies, we tested how well macroscopically recorded neuronal activities, i.e., ECoG evoked signals, could also predict microscopically recorded activities, i.e., spikes in a visual-task condition. In the ECoG data, the transmission delays are given as the time delays of the primary peak in the individual time series of evoked activities that occurred at the target region within 100 ms of stimulus onset via the primary visual region (Fig. 1A). This is because past studies have mentioned 100 ms is enough to reach visual information from occipital primary region to frontal pole in macaque brains^33^.

**Figure 2:**
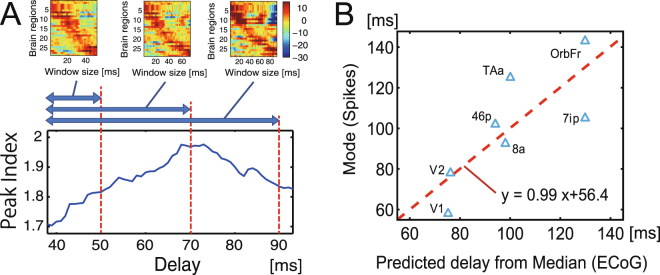
Comparison of delays between neuronal spikes and ECoG within stimulus-driven activities. The bottom section of panel A shows the relationship between the length of the time window used to select peak points of cross correlations (the x-axis) and the averaged *Peak Index* of the waveforms of all electrodes (y-axis). In order to visually observe the traveling waves of visually evoked ECoG signals, we also show the upper panels in panel A. Here, the x-axes indicate the size of the time windows and the y-axis is the index of brain regions expressed in terms of ECoG sensors. The values on the y-axis were sorted according to the time delay of the peak point. These three panels correspond with three different time windows, 0–50, 0–70 and 0–90 ms. Panel B shows the results of the main predictions of delays of visually evoked electrical spikes (y-axis) by fitting a linear model to the ECoG signals (x-axis).

Next, we evaluated the sharpness of the averaged waveforms using a variable named *Peak Index* (Fig. 1B) because we could expect that responses in brain regions directly connected through anatomical pathways would be sharper than responses in indirectly connected brain regions. The upper small panels in Fig. 2A are contour maps of averaged voltages, which were measured ECoG sensors locating on various brain regions (Table 1). The diagonal pattern of three contour maps confirm the presence of a clear flow of electrical activity expanding to whole brain regions, and indicate that the flow is stable against changes in the size of the time window used to search for peak points. However, if the time window is too long, it is possible to erroneously select indirect or separated pairs of brain regions. Equally, if the time window is too short, long connections, which have long propagation times, may be ignored. Therefore, the relative confidence of reconstructed electrical flows, quantified by the averaged Peak Index, should depend on the size of the time window. As shown in the main panel in Fig. 2A, the averaged Peak Index was maximized when we searched for peaks in a time window that was 70 ms or shorter.

**Table 1:** Summary of brain region labels in four monkeys.

ECoG channels	Su	Georg	Kin2	Chibi
1	NONE	2	NONE	7b
2	NONE	7b	NONE	7b
3	NONE	7op	NONE	7op
4	NONE	Toc (PA)	9	7a
5	NONE	12	M2 (6 M)	46p
6	9	6Vb	M2 (6 M)	45
7	9	6Vb	M2 (6 M)	6Val
8	M2 (6 M)	6Vb	6Dc	4c
9	NONE	2	NONE	1
10	NONE	7b	NONE	2
11	NONE	7b	6Ds	7a
12	46p	7op	6DR (6D)	7a
13	6Ds	7a	6DR (6D)	V4
14	6Ds	12	6Dc	NONE
15	6DR (6D)	46p	4	46p
16	6Dc	45	4	6Ds
17	12	6Val	12	4
18	NONE	2	NONE	4
19	46 v	5 v	6Ds	1
20	46p	5 v	6Ds	2
21	6Ds	7b	6DR (6D)	5D
22	6Ds	7a	6Dc	7a
23	4c	12	6Dc	NONE
24	4	NONE	4	12
25	2	NONE	4	NONE
26	12	46p	46p	46p
27	46p	46p	46p	6DR (6D)
28	45	6Ds	6Ds	4
29	6Val	4c	4c	4
30	4c	2	4	1
31	2	2	4	1
32	2	5 v	2	5D
33	2	7a	2	5D
34	5 v	NONE	7a	NONE
35	5D	NONE	7a	NONE
36	6Vb	46p	46 v	NONE
37	6Vb	6Ds	46p	6Ds
38	6Val	6DR (6D)	6Ds	6DR (6D)
39	6Val	6DR (6D)	4c	6Dc
40	2	6Dc	4	4
41	5 v	4	4	4
42	5 v	2	5 v	1
43	7b	5D	5 v	1
44	7a	5D	7a	5D
45	7a	NONE	7a	NONE
46	DP	NONE	V4	NONE
47	6Vb	NONE	46p	NONE
48	6Vb	9	45	9
49	6Vb	9	6Val	9
50	6Vb	M2 (6 M)	6Val	M2 (6 M)
51	2	M2 (6 M)	2	M2 (6 M)
52	5 v	M2 (6 M)	2	M2 (6 M)
53	7b	M2 (6 M)	5 v	M2 (6 M)
54	7op	6DC	7b	6DC
55	7a	4	7a	4
56	PrCO	NONE	12	NONE
57	PrCO	NONE	6Vam	NONE
58	6Vb	NONE	6Val	12
59	S2	24b	6Val	24a
60	S2	24d	2	24d
61	7op	24d	2	NONE
62	Toc (PA)	23	7b	3a
63	V4	10 m	7b	NONE
64	V4	10 m	7op	10 m
65	6Vb	NONE	7a	NONE
66	PrCO	12	6Vb	NONE
67	PrCO	PrCO	6Vb	NONE
68	S2	TS (ST)	6Vb	NONE
69	TS (ST)	TS (ST)	6Vb	TS (ST)
70	Tpt	TAa	7b	TS (ST)
71	V4	TE1–3	7op	Taa
72	V4	TE1–3	Toc (PA)	TE1–3
73	V1	Vot	7a	Vot
74	TS (ST)	V4	6Vb	V4
75	TS (ST)	VP	6Vb	V2v
76	TS (ST)	V1	6Vb	V1
77	TAa	V1	S2	V1
78	TAa	V1	S2	V1
79	Vot	V1	Tpt	V1
80	Vot	PrCO	Tpt	NONE
81	V4	PrCO	V4	TS (ST)
82	V1	TS (ST)	V4	TS (ST)
83	V4	TAa	V4	TS (ST)
84	V2d	Vot	V4	Taa
85	V2d	V4	V4	Vot
86	V2d	V4	V2d	V4
87	V1	V2v	V4	V2v
88	V1	V1	V2d	V1
89	V1	V1	V2d	V1
90	V1	V1	V2d	V1
91	V1	V1	V1	V1
92	V1	NONE	V1	6Vb
93	V1	NONE	V1	6Vb
94	V1	TS (ST)	V1	6Vb
95	TS (ST)	TS (ST)	PrCO	2
96	TAa	TS (ST)	PrCO	S2
97	TAa	V4	TS (ST)	Tpt
98	TE1–3	V4	TS (ST)	Tpt
99	Vot	V4	TAa	V4
100	V4	V1	V4	V4
101	V4	V1	V4	V1
102	VP	V1	V1	V1
103	V1	V1	V1	V1
104	V1	V1	V1	V1
105	V1	6Vb	V1	6Vb
106	V1	6Vb	V1	6Vb
107	NONE	6Vb	12	6Vb
108	NONE	S2	6Vb	2
109	TS (ST)	TPOc	PrCO	TPOc
110	Taa	V1	TS (ST)	V4
111	TE1–3d	V1	TS (ST)	V4
112	TEa	V1	TAa	V1
113	Tea	V1	TE1-3	V1
114	Vot	V1	TE1-3	V1
115	V4	V1	Vot	V4
116	V4	V1	V4	V2d
117	VP	V1	V4	V1
118	V2v	V1	V1	V1
119	V1	V1	V1	V2d
120	V1	V1	V1	V2d
121	V1	V1	V1	PIP
122	NONE	V2v	NONE	PIP
123	NONE	V1	NONE	PIP
124	TAa	V2v	NONE	NONE
125	TE1-3d	V2d	TAa	5D
126	TE1-3d	V2d	NONE	31
127	TE1-3	V2d	NONE	2
128	NONE	V2d	NONE	2

From left to right, the first column shows ECoG channels from the Neurochyco database. The second–fifth columns contain indexes of parcelled structural brain regions located under the ECoG sensors^28^. We compared the locations of ECoG channels and parcelled regions using Caret software^88^. Because the locations of the ECoG sensors were different among individual monkeys, the structural regions vary among them. (OrbFr: Orbital prefrontal cortex, PreM: Pre-Motor Cortex, M1: Primary Motor Cortex, SEF: Supplementary eye field, 5: Area 5, 8 A: Prefrontal area 8 A, 7ip: Parietal area 7ip, TA: Temporal anterior region, TAa: Area TAa, TPO: Temporal parietal occipital, V4: Visual area 4, V2: Visual area 2, V1: Primary visual area).

As shown in the upper panels in figure A, when we select 0–70 ms as the time window, we could visually observe a relatively sharper and clearer uni-directionally propagating wave of cortex. Because this result infers that this time window is optimal for detecting the clearest flow of electrical activity through the macaque cortex, we determined the time delays for individual pairs of brain regions in the time window 0–70 ms.

Figure 2B shows the scatter plot of time delays in neuronal spikes (y axis), which are defined as mode values of firing rates locked to the visual stimulation, and predicted delays of spikes from evoked ECoGs (x-axis) according to a linear regression model (y = ax + b). In the regression model, the gradient value was close to 1, indicating that we selected an appropriate time window. Therefore, we searched for activity peaks at 35 (40–75) ms, starting from the primary visual area: A time delay of 40 ms was previously estimated as the time required for visual information to travel from the retina to the primary visual region^33^.

### Comparison between neuronal spikes and ECoG in the task-free condition

So far, we have related visually evoked ECoG dynamics to spike-based latencies evoked by visual stimuli. Next, we sought to determine how well the ECoG signal flow during the Anesthetized and Awake Task-Free conditions, which had no clear visual stimulus onset, could reproduce the time delays recorded as electrical spikes (see subsection 1 in Materials and Methods). The relationship between evoked and spontaneous activity is a fundamental issue in neuroscience^34,35^. Even in non-stimulated conditions, our brains are always working to process various cognitive information including visual and motor information internally^36,37^.

In the Anesthetized and Awake Task-Free conditions, we estimated the time delays in three steps (Fig. 6, subsection 4 in Materials and Methods): First, we adopted the peak delays of cross-correlations between two time series at two brain regions (see subsection 3 in Materials and Methods) as the time delays for signal transmission on a direct pathway connecting the two brain regions. Second, we summed the delays necessary for all individual path steps along the pathway. The example in Fig. 7 shows pathways connecting region I to region J. Third, we calculated the weighted average of all delays for all pathways based on the three different weight models.

**Figure 3:**
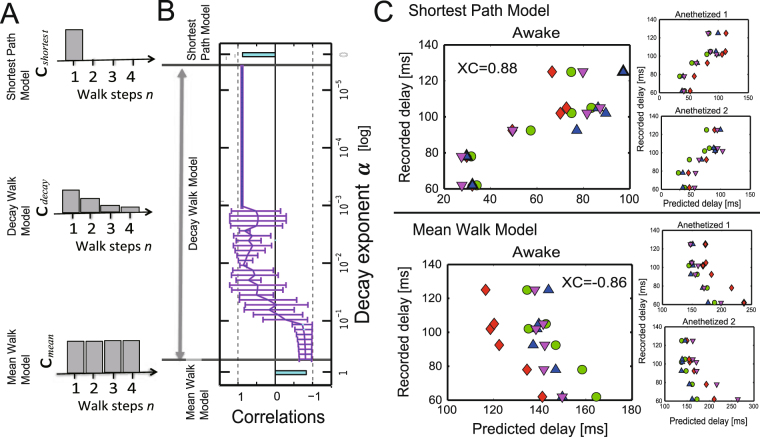
Predictions of time delays of electrical spikes from ECoG data under the no-task condition. Panel A shows histograms of the weights used to prepare the three Walk Ensemble Models. From top to bottom, the histograms show the weights for the Shortest Path (SP) Model, the Decay Walk (DW) Model, and the Mean Walk (MW) Model. Please notice the length of shortest paths are not always 1. Panel B shows the correlations between delays of neuronal spikes and delays predicted using the three models. The top and bottom bars are correlations for the SP and MW model, respectively. In the MW Model, the mean Walks were calculated from samples included in the one-sigma window (mean value ± one standard variation). The intermediate line on the y axis between the two models corresponds to the correlations for the DW model, determined as a function of exponent *α* in the equation *c*_*n*_ = *α*^*n*^. In panel C, the upper three scatter plots show the results for data processed with structural constraints based on the SP model, and the lower three panels are scatter plots for the MW model. In both cases, the biggest panels are the results under the awake task-free condition, and the two small panels show the results for the two anesthetized conditions. The four different markers indicate the four different individual monkeys.

The three “Walk Ensemble Models” (Fig. 3A or Fig. 7B), which determine how to add individual time delays along selected chains of edges in three different ways, lead to completely different trends. The chain of edges referred to as a *Walk*, a graph-theoretic concept, is a set of nodes connected successively by links such that connecting back to the same node is allowed. Interestingly, time delays predicted from the Shortest Path (SP) Model showed a clear positive correlation with spike timings, while the Mean Walk (MW) Model showed a clear negative correlation. The SP Model gives excessively high weights to walk ensembles holding the shortest paths. Inversely, the MW Model gives excessively high weights to ensembles holding relatively longer Walks because the number of samples holding relatively longer Walks is exponentially larger than the number of samples holding relatively shorter Walks. Therefore, we designed an intermediate model, the Decay Walk (DW) model, using weights that decay exponentially depending on the increase in Walk *n*. For example, if the transmission probability decreases by *α* (0 < *α* < 1) per one walk step, the multiplied transmission probability for *n* steps of Walk would be expressed as *c*_*n*_ = *Prob*(*α*,*n*) = *α*^*n*^(0 < *α* < 1). This corresponds with the natural probability expressing how often individual walks will be used in a random walk process. Depending on the index *α*, the DW model gradually changes from behaviour similar to the SP model to that similar to the MW model. If *α* = 1, the DW model corresponds with the MW model, and for the limit *α* → 0, we expect the result to approach that of the SP model.

Interestingly, we found that at an intermediate *α* in the DW model, the correlation between the spike delays and the delays estimated from spontaneous ECoG under the constraint of structural connectivity, reversed from a strong positive to a strong negative value (Fig. 3B). The scatter plots between the original spike delays and estimated delays show natural diagonal distributions at strongly correlated regions (Fig. 3C). At the intermediate phase (10^−2^–10^−1^), the correlation gradually changed between these positive and negative values. This result indicates that the first electrical signals (<100 ms) to reach their destinations in brain networks use ensembles of shorter paths more often than ensembles of longer paths. It seems that the exponent *n* must be sufficiently smaller than 10^−1^ in the weight *α*^*n*^. In subsection 4, we will address what may have determined the transition point.

### Time delay for spatial spreading and conduction velocity

**Figure 4:**
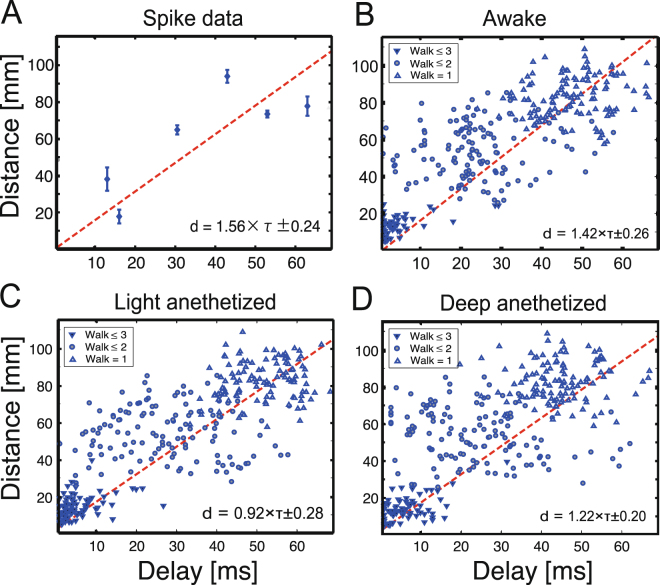
Estimating conduction velocities on the cortical connectome at three conscious levels. Panel A contains a scatter plot showing distances between pairs of brain regions vs necessary delays to transmit neuronal spiking activities between them. Panels B–D show three dense scatter plots of the relationships between distance and necessary delays, estimated from the ECoG data. The three panels B-D reflect data for different cognitive states (awake state, light/deep anesthetized states). In the three panels, the downwards-pointing triangle markers indicate the results for directly connected paths (Steps of Walks *n* = 1), circles denote the results for samples with *n* = 2, and upwards-pointing triangle markers correspond to samples with *n* = 3. The inserted equations in the individual panels are equations for fit lines (d: distance, *τ*: delay). The two dotted lines in each panel are fit lines for samples with Walks that contain less than 4 steps. When the distance between two brain regions is longer (y-axis), the transmission requires more time (x-axis). In all states, the conduction velocity (the slope of the fit line) was ~1.0–1.5 m/s.

So far, we have not observed spatial dimensions. Because brain regions are embodied in space, the spatial coordinates should also reflect the temporal dynamics of propagating electrical signals. Therefore, we examined the relationships between the spatial distances summed along the walk steps from the “Origin” to the “Target” brain regions, and the necessary delays for electrical propagations through these walks (Fig. 4). Refer the detailed analysis scheme to Fig. 7. From this relationship between distances and delays, we can estimate conduction velocity of electrical brain signals in the brain. Although the conduction velocity of electrical brain signals is a fundamental question in neuroscience, it has not yet been possible to estimate the velocity from brain-wide observations due to limitations in past technology or existing data. Here, we successfully estimated the velocities for three conscious or arousal levels as ranging from 1.0–1.5 m/s. Interestingly, the estimated velocity was fairly close to the conduction velocity estimated from the perspective of optimally synchronous brain states in a computational simulation study^38^. We also found that the velocities were only slightly different (not significant) among the different conscious levels (p > 0.3, panels B–D in Fig. 4).

### Walk ensemble models and Communicability

In section 2, we reported that the time delays in firing spikes could be successfully estimated from ECoG data when we considered the cases in which shorter Walks (with structural constraints) are used more frequently than longer Walks. The relative frequency of use between shorter and longer Walks seems to be characterized by *α* in the Delay Walk (DW) model. Thus, our final topic in this report involved determining the transition point according to *α*.

**Figure 5:**
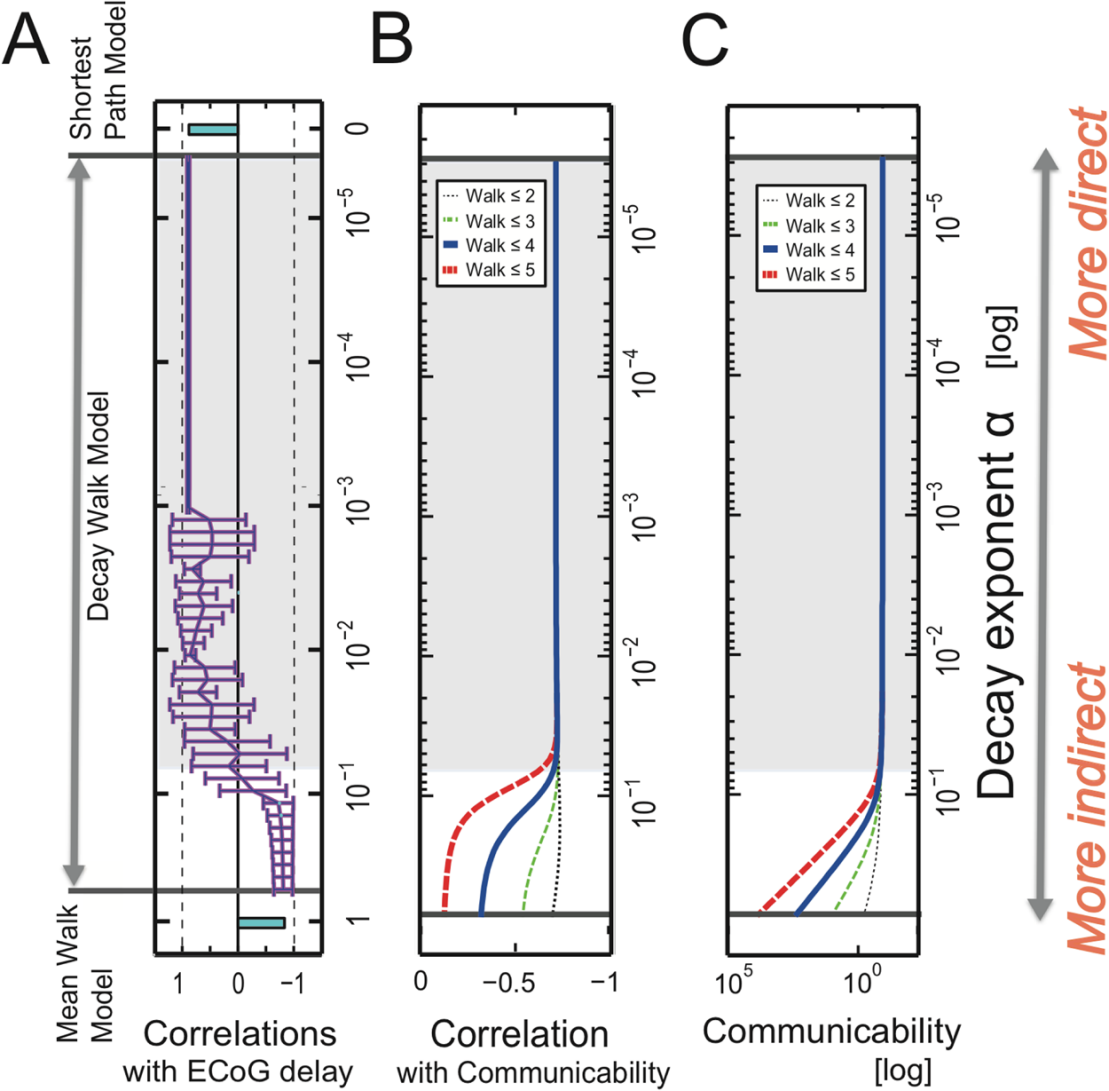
Predictions of time delays of electrical spikes from Communicability. Panel A is the same figure from panel B in Fig. 3. The correlation between time delays recorded by electrical spikes and the estimated time delays from the ECoG data is described as a function of the decay factor *α*. Panel B shows the correlations between Communicability and the delays of the electrical spikes. The four lines correspond to cases in which the maximum Walk steps were limited to 2, 3, 4, and 5 steps. Panel C shows the original Communicability as a function of *α*. The four lines have the same meaning as those shown in panel B.

The DW model has exactly the same form as a class of novel measure: the Communicability between two nodes of a network. This was introduced in a series of studies of complex networks^31^ [see also equation (15) in ref.^32^]. Importantly, *Communicability can systematically quantify how non-shortest paths/walks contribute to the spread of information in many systems, including the brain*. Let *A* denote the adjacency binary matrix of the network; each element *A*_*ij*_ is one if a node *i* is linked to a node *j*, and is zero if not. In order to remove influence of self-loop connections, we set all diagonal components to zeros. The *Communicability* between nodes *p* and *q* can be defined as the (*p*, *q*) element of the matrix $G=\sum _{n=0}^{\infty }{c}_{n}{A}^{n}$. The (*p*, *q*) element of the summand (*A*^*n*^)_*pq*_ is equal to the number of Walks that connect nodes *p* and *q* in *n* steps. The Communicability *G*_*pq*_ therefore takes account of each *n*-step Walk with the weight *c*_*n*_. The weight can be *c*_*n*_ = *α*^*n*^ with a small parameter *α*, for which *G* = (*I* − *αA*)^−1^, or ${c}_{n}=\frac{{\beta }^{n}}{n!}$, for which *G* = *e*^*β.A*^. To more systematically understand the given results for our neurophysiological data, we also calculated Communicability as a function of the decay factor *α*. Figure 5B is the correlation between Communicability between a pair of brain regions and the necessary delays required to transmit neuronal spikes between them. We could observe a phase change for the correlations of spike delays with Communicability around the similar region of *α* ∼ 0.07. For reference, 5-A reproduces Fig. 3B, which showed the correlation between the necessary delays and the Delay Walk Model. The light grey region (*α* ≲ 0.07) in Fig. 5A correspond with the region where positive correlations were observed, and negative correlations were observed at the white bottom region (*α* ≥ 0.07). We can find that the phase change happened around a similar region *α* ∼ 0.07. The original Communicability is also shown as Fig. 5C. At the region where correlations were stable, Communicability also held a stable value. So, these phase changes of correlations seem to be captured by the modulation of “Skelton” structural network itself, and by the well-organized measure Communicability. Besides, the phase change of correlations between spike delays and Communicability was found in all cases when we limited the maximum number of Walk steps to 3–5, although the trend changed when we limited the maximum Walk steps to 2 (Fig. 5B). This result indicates that at least 3 steps of Walk should be considered to properly characterize the relative frequencies of use between the shorter and longer paths.

## Discussion

### Main findings

This study produced four main findings: First, we demonstrated that ECoG signals can be used to predict the timing of evoked electrical neuronal spikes elicited by visual and auditory stimuli. Second, we confirmed that spontaneous ECoG under a blindfold condition (without stimuli triggers), can predict the timing of visually evoked neuronal spikes. The prediction performance from the blindfold data was efficiently supported by structural constraints. Third, we estimated the propagation velocity (conductance velocity) as 1.0–1.5 m/s using connectomic data, and found that the velocity does not depend on conscious level. Fourth, we demonstrated that Communicability can be used to systematically characterize the contributions of the shortest paths and non-shortest paths in the general pattern of transmission delay.

### Multi-scale neuronal recording technologies

**Figure 6:**
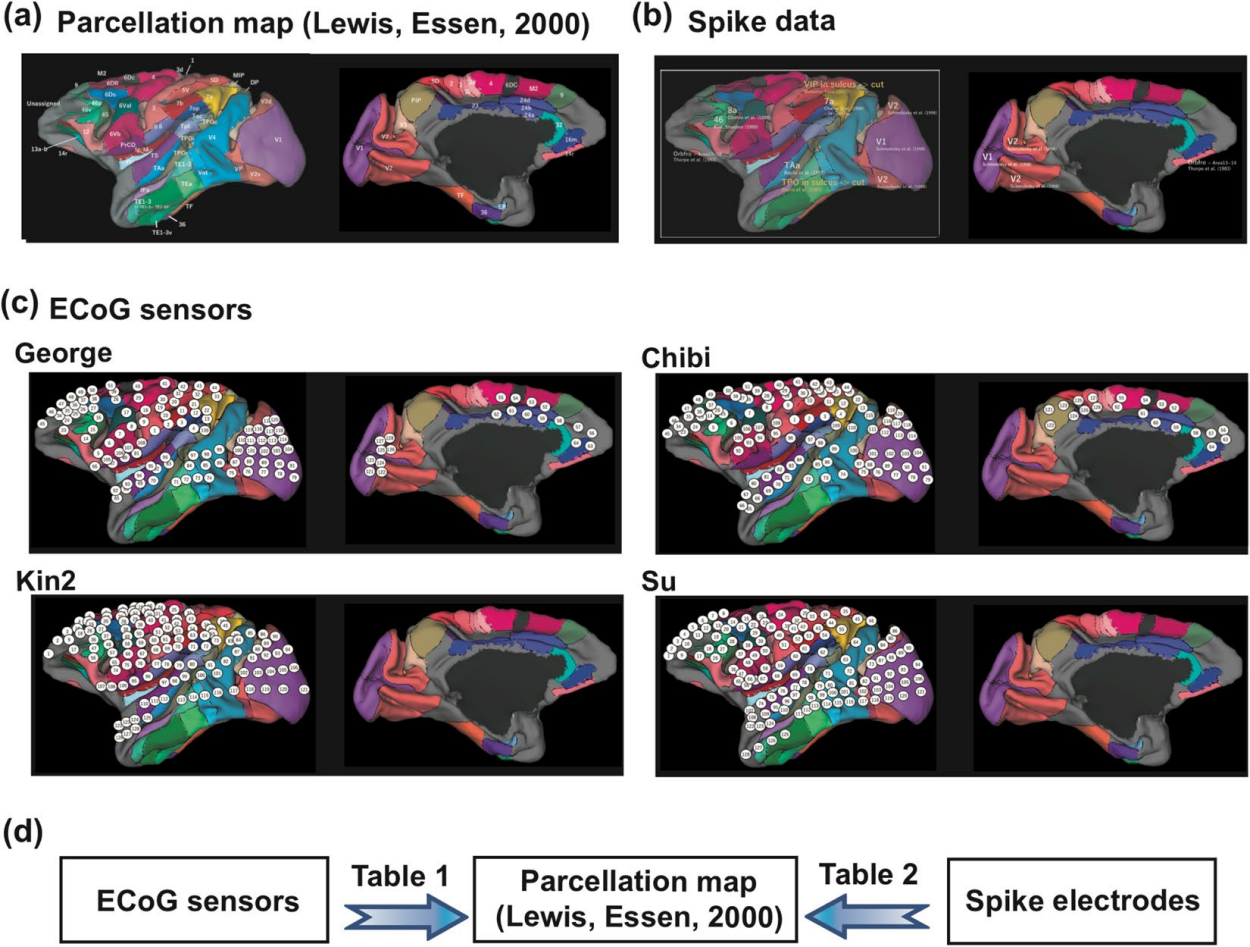
Spatial maps of anatomical percolation, neuronal spike data, ECoG sensors. (**a**) We selected the scheme defined in Lewis, Essen (2000)^72^ as the parcellation map of cortex here, and visualized the maps using Caret software^88^. (**b**) This spatial map shows the distribution of recording spots of spike data listed in the Table 2. (**c**) Distributions of ECoG sensors adopted in Neurotycho data set. (**d**) We separately performed two co-registrations between ECoG sensors and the anatomical parcellation map as listed in Table 1, and between recording spots as spike data and the anatomical parcellation map as listed in Table 2.

We demonstrated predictions of the time delays of visually evoked spikes from ECoG data recorded in the blindfold condition because spikes are primarily important for information processing of the brain (Fig. 6). Surprisingly, the slope for the regression plot between ECoG and electrical spikes was 1. This result indicates that latency is a robust feature between these completely different two recording modalities. Previous studies have successfully predicted spatial patterns of functional spontaneous activities observed from fMRI using spatial patterns of structural networks from diffusion tensor/spectral Imaging^13,39,40^. The temporal resolution of fMRI is longer than one second. To contrast this, we aimed to show how high structural constraints could influence the determination of temporal dynamics of neuronal spikes using a higher temporal resolution signal, i.e., ECoG, which is less than a millisecond.

Because the spatial scale recorded in ECoG, 1 cm, is over 100 times the spatial scale recoded in LFP, 1 mm, and many complex spatial patterns can be generated in the spatial map, the success of prediction using ECoG signals is a non-trivial result. Additionally, past predictions using LFP tested limited brain regions, while the data set used in our study included whole cortical networks. With knowing gaps between SUA and LFP, the positive correlation between pre-synaptic effects and post-synaptic effects is a presumable physiological phenomenon because if electrical signal at pre-synaptic neuron increases, spikes of post-synaptic neurons will also tend to increase. In fact, several studies have reported high prediction performance of spike timings from Local Field potentials (LFPs)^41–43^. Therefore, the main non-trivial results of the present study would be given insight into the integration among different spatial scales at millisecond-order temporal dynamics^44^.

### Subcortical contributions

When simultaneously observing many brain regions, it is important to consider the important roles that subcortical regions play in mediating electrical interactions between cortical regions^45,46^. Indeed, how cortico-subcortico-cortical connections and subcortical pathways influence global dynamics within the cortex is an interesting question for future research^47,48^. Collaborative studies involving simultaneous recordings from many subcortical and cortical regions will improve our current understanding of neuronal signal transmission^49^.

### Transmission delay and Communicability

From comparison among the Shortest Path, Decay Walk, and Mean Walk Models, the Shortest Path Model provided high prediction performance of spike data although the relatively simpler expression than the Decay Walk Model. In other words, the Shortest Path Model seemed to be approximately a good model of communication dynamics in the brain. Besides, from results when increasing α in Delay Walk model, we could also estimate how much non-shortest paths, or non-direct paths, can contribute to the communication dynamics in the brain. In fact, the correlation between neurophysiological delays and estimated delays was inverted from positive to negative aroundα~0.01–0.05. Furthermore, Communicability significantly increased with the increase of α around the same region (Fig. 5C). Because Communicability is an excellent measure quantitatively evaluating the contributions of non-direct paths, we would be possible to say the relative percentage of contribution of shortest path is 95–99%. In general cases, we also need to notice carefully that slow components, such as P300^44,50^, because they may reflect activities where the non-shortest paths are more frequently selected. Here, although we could not completely check the mechanism why the negative correlation happened because of high computational demands, we estimate the negative correlation may have happened because of the cut off of path lengths in the Decay Walk model.

Communicability has been previously applied to weighted brain data collected via diffusion tensor imaging^51^. Furthermore, Communicability has been found to be a sensitive measure for quantifying changes in brain regions remote from Stroke foci in both an experimental study^52^ and a computational simulation^53^. The removal of nodes with high Communicability, as well as the removal of rich-club nodes, can severely impact global communication in the brain^54^. In the near future, these knowledges will be continuously connected together.

### Estimation of transmission velocity

We also estimated propagation velocity. With respect to past studies, our main novel contribution is that we evaluated the propagation velocity on a global brain scale. Indeed, past studies estimated propagation velocity of neuronal spikes within limited brain regions^55–58^. In a previous computational modelling study, propagation velocity was estimated in terms of the optimality of synchronous activations between brain regions^54^. In our study, the propagation velocity was fairly stable, even at different Conscious levels. Note that the variance or higher statistical moments of the velocity could potentially describe the differences between the Conscious levels (Fig. 4). In general, what is a characteristic of conscious level is an interesting scientific question^59–61^. Meanwhile, we could use more causal measures, such as Transfer Entropy, instead of non-causal measures, such as cross correlations. People may expect that Transfer Entropy can improve the current functional connectivity results better than Cross Correlation, as it will also clarify the relationships between functional connectivity and structural connectivity^17,62^. However, notice, the problems of causality are not so serious issue in this study because we also directly included structural constraints into the analyses process.

The estimated velocity contained clear variability, and the general form of the histogram of the propagation velocity followed a gamma distribution^63^. A physiological interpretation of the histogram form is a potential topic for future studies. Anatomical connectivity also contains variability in terms of connection strength, and recent studies have reported that there are more weak connections than previously presumed^64^. Physiologically, axonal conduction delays can vary widely depending on myelination or demyelination^5^, axon diameters^65^, and the density of sodium channels^66^, and also depend on the forms of dendritic branches and cell types. Notice that the globally most influential or typical velocity is very important even a wide variety of velocities exist. Future work evaluating the variety of propagations^67^ in brain-wide distributions^7,68^, and considering the detailed synaptic^52,67,69,70^ and informatic^8^ topologies of neurons will contribute greatly to this field.

### Final remarks

This study focused on the time-delay in cortical communication dynamics. Using Communicability and the index *α*, we quantitatively evaluated how a relative frequency of use between shorter and longer paths influences the information flow in the unified theoretical framework. How the human brain evolved such an efficient network organization with the selective use of shorter paths remains an interesting essential question. Shorter paths reduce wiring cost, while some long paths are unavoidably necessary for the integration of information. Therefore, it is important to consider both the optimality and efficiency of the brain structure. We believe that our results represent an important step in generating increasingly realistic predictions of brain dynamics.

## Methods

### Data acquisition

Using a neuroinformatic approach, we combined three data sets acquired using different modalities by independent research groups: (1) spike-based visual responses in single-unit recordings, (2) brain-wide field dynamics recorded with ECoG, and (3) anatomical connectivity network data among cortical regions from tracer injection studies. The ECoG data provides the electrically propagating signals, the anatomical connectivity data provides constraints of propagating pathways, and spike data is used to compare with ECoG data. All data were collected from the macaque cortex, and processed using the following methods:

**First**, we obtained macroscopic functional data, specifically ECoG data from four macaque monkeys, from the Neurochyco database^26,51,71^. The excellence of this data is that the ECoG sensors cover almost all cortical surface. This allows us to characterize the global electrical propagations. The data set includes data recorded continuously from monkeys that were blindfolded and not engaged in any specific tasks, i.e., the “Awake Task-Free condition”. ECoG recordings from anesthetized monkeys are referred to as those collected in the “Anesthetized condition”. The data set also included a visual stimulation experiment. In the visual experiment, a grating stimulus was presented around a fixation cross with one of eight randomly selected grating orientations. The stimuli were shown for 2 second in every trial. Refer to the web page (http://wiki.neurotycho.org/Anesthesia_Task_Details) for more detailed information about the ECoG experimental procedure.

**Second**, to optimally model the transmission pathways of electrical signals between brain regions, we considered the constraints of underlying structural networks. We prepared the structural networks of the monkey brain based on the data given in Lewis and Van Essen (2000)^72^. In their model, the networks also cover entire cortical regions, and include the strengths of connections, discretized into seven levels. This atlas is shared publicly in the CoCoMac database^73–75^. This database has contributed to many investigations, including a comparison between monkey and human brains^76^, assessment of the relationship between structure and function^77^, and relationship between network architecture and cognition^78^. Currently, this database is continuously maintained as the Scalable Brain Atlas^79^.

**Third**, we prepared a summary of responding peak latencies of neuronal spikes from past neurophysiological studies in order to support the neuronal basis of macroscopic ECoG signals. We assessed neuronal spike timings associated with visual information processes for not only occipital visual areas, including V1 (primary visual) and V2 areas^80^, but also temporal areas such as TPO and TAa^81^, parietal areas including area 7ip^82^, frontal areas such as areas 8a, 46^82,83^, and the orbitofrontal region^84^. Several previous articles have reviewed trends in the time delays of visual evoked activities^33,85^. The peak latencies of neuronal spikes were represented by the mode values of firing pattern histograms. Note that, although many other studies have recorded evoked firing activities, we selectively used the data sets to those that recorded from the cortical gyri. This is because the ECoG data, which will be compared later, was recorded only from gyri. Additionally, if we could not extract mode values (peak points) from figures given in past reports, we excluded that data from our analysis.

### Integration of data

To transform the original structural network data into a network with the spatial resolution of the ECoG sensors, we labelled groups of ECoG sensors according to individual brain regions by co-registering given sensor positions of ECoG sensors provided in the Neurochyco database onto a spatial parcellation scheme of the monkey cortex^72^ using Caret software^86^ [Fig. 6c,d]. Then, ECoG sensors and spike electrodes are also separately co-registered with the parcellation map [Fig. 6b,d]. Table 1 shows the list of sets of 128 ECoG sensors’ indexes and the names^72^ in the structural segmentation for individual monkeys. Because the locations of the ECoG sensors were different among monkeys, the corresponding structural brain regions also varied (Right four columns in Table 1). Here, the cortical regions at the sulci or on the longitudinal fissure were eliminated because the ECoG sensors were not indwelled at those regions. The names of the structural brain regions corresponding with the indexes are separately summarized in Table 2. The similar comparison process was used in our previous study^87^. Because several regions were eliminated in this process, we regarded pairs of nodes, connected through one intermediate node, as connected. This pre-processing improved the prediction performance of spike timings of neurons^35^.

**Table 2:** Summary of spiking neuron data.

Region name	Number of neurons	Reference	LV00
V1	74	Schmolesky *et al*.^26^	V1
V2	61	Schmolesky *et al*.^26^	V2
TAa	98	Baylis *et al*.^81^	TAa
TPO	547	Baylis *et al*.^81^	TPOr, TPOc, TPOi
7ip	94	Chafee *et al*.^82^	7a, 7b, 7op
46	62	Kim, Shadlen^83^	46p
Orbitofrontal	494	Thorpe *et al*.^84^	10m

The first column lists the abbreviated names of brain regions that could be used to assess neuronal spikes. The second–third columns are number of neuron recorded in these brain regions, and their original reference articles. The last forth column is names of parcelled structural brain regions corresponds with the brain regions used for neuronal spike recordings. LV00 is an abbreviation of the parcelled map used in ref.^72^.

### Estimation of time delays of neuronal spikes from visual stimulus-evoked ECoG activitis

In the visual stimulation experiment, we estimated the transmission delay based on the time of the primary big sharp peak of evoked potential after visual stimulation. Notice that the structural networks were not necessary to determine delays for evoked activities, and that they were used only to estimate delays for spontaneous activities. As a pre-processing, we averaged the 210 trial data points after subtracting the 50 Hz component using a notch (band cut) filter with a 5 Hz standard deviation to eliminate power supply noise. Then, we selected the largest peak point between 0–Tms (T <100 ms) after the stimulus onset (Fig. 5B), and used the time delay of that peak point as the delay of the ECoG evoked data. We explain how the time delays for the anesthetized conditions were extracted in subsection 3 in this method section. Then, *we evaluated the sharpness of the averaged waveforms* using a variable named *Peak Index* (Fig. 1B) to extract the most optimal time window. The Peak Index was mathematically defined by the following equation:

1 $${\rm{Peak}}\,{\rm{Index}}=(({{\rm{ECoG}}}_{{\rm{\max }}}(i)-{{\rm{EoG}}}_{{\rm{\min }}}(i))/\delta (i))({\rm{i}}:\,{\rm{index}}\,{\rm{of}}\,{\rm{ECoG}}\,{\rm{sensors}})$$

Here, ECoG_max_ (*i*) and ECoG_min_ (*i*) are the maximum and minimum values, respectively, of the ECoG signal recorded by sensor *i*, and *δ*(*i*) is the standard deviation when we fit the data to a Gaussian function around the primary peak point. Therefore, this index evaluates the average of amplitudes at the ECoG peaks for all sensors with sigma *δ*(*i*) as the unit. This value was averaged for all sensors involved in individual brain regions to get an averaged Peak Index representing interactions between brain regions.

### Estimation of time delays from non-time locked ECoG activities along structural paths

**Figure 7:**
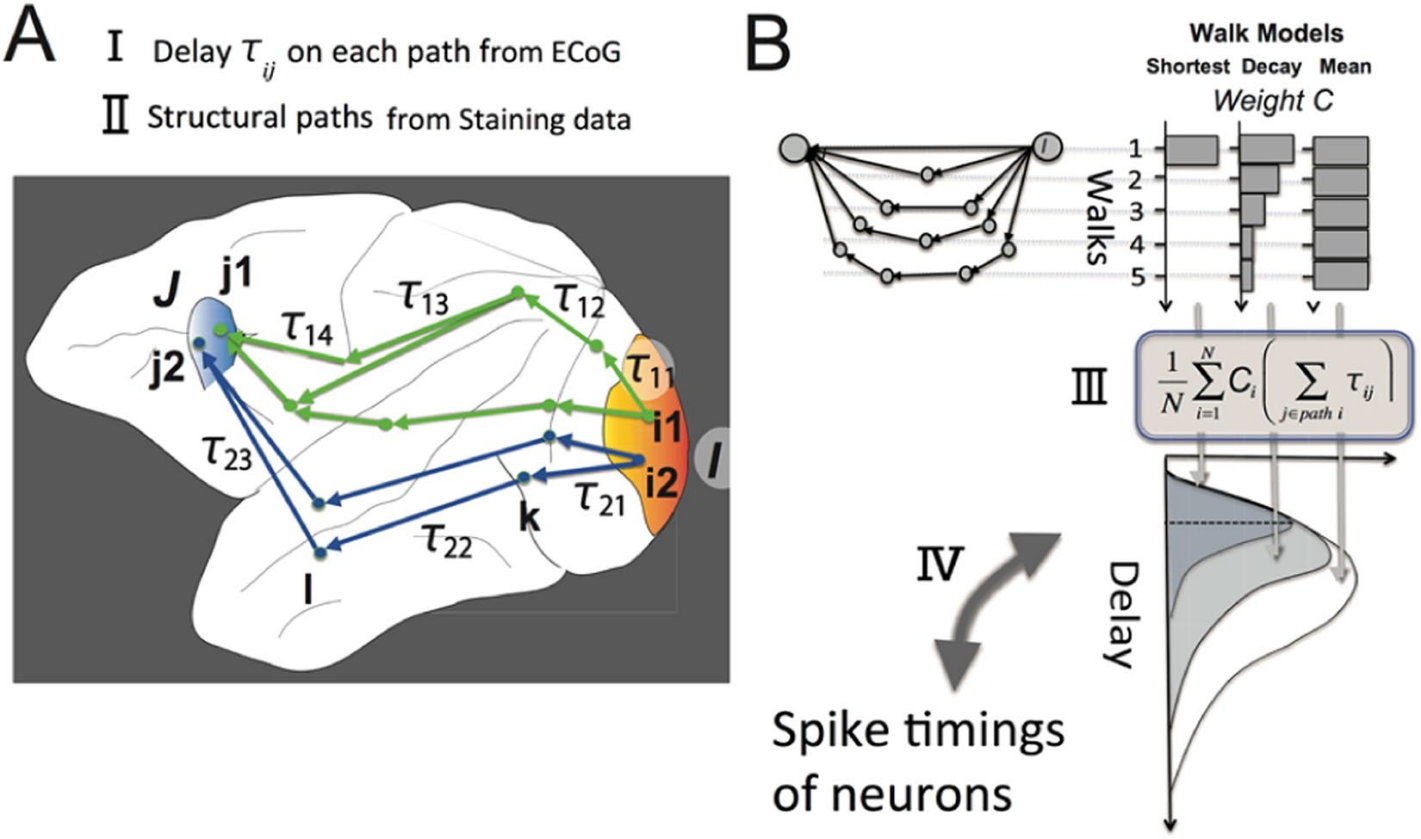
Predictions of time delays of electrical spikes from ECoG data in the Task-Free condition. (**A**) Our scheme involves calculating the time delays from a starting region I to a goal region J using spontaneous activities and structural pathways. In the present study, we used a cortical parcellation scheme according to Lewis and Van Essen (2000)^72^. Regions I and J represent one of the pairs of 98 cortical regions included in the parcellation scheme. There are several ECoG sensors on both regions I and J. Here, we simply consider only two sensors, i1 and i2 (j1 and j2), to exist in each region I (J). In this example, the pathways from region I to region J involve all four combinations of paths from sensors on the region *I*, i1 or i2, to sensors on the region J, j1 or j2. Each combination of the starting and goal points may involve many paths (Steps of Walk n ≤ 4). Each delay *τ*_*ij*_ at a step j on a path i was given as the peak delay within 100 ms (stepI). In this panel A, for example, if the activity is transmitted using the most dorsal pathway, the total delay is ${T}_{1}=\sum _{p=1}^{4}{\tau }_{1p}$, and if the activity is transmitted on the most ventral pathway, the total delay is ${T}_{2}=\sum _{p=1}^{3}{\tau }_{2p}$. (**B**) Now, in general, we have time delay $\sum _{p=1}^{{n}_{q}}{\tau }_{qp}$ for every possible structural pathway (step II). Here, *τ*_*qp*_ is a delay necessary to transmit electrical activities on a *p*th step on a *q*th pathway. Therefore, the total step on the *q*th pathway is *n*_*q*_. *Notice that we now do not care about the difference of adjacent ECoG sensors if they are involved in the same cortical region*. We then prepared three models to calculate the weighted averages of time delays $\frac{1}{{\sum }_{q=1}^{N}{{\rm{C}}}_{q}}\sum _{q=1}^{N}{{\rm{C}}}_{q}(\sum _{p=1}^{{n}_{q}}{\tau }_{qp})$ given from many *N* pathways connecting region I to region J. *C*_*q*_ indicates the relative weight given for the *q*th pathway. Essentially, how the weights depend on *Walk* steps is expressed using three models: The three bar graphs in the top-right show the relative weights for individual Walks for the three models. From left to right, the bar graphs correspond to the *Shortest Path* (SP) model, *Decay Walk* (DW) model, and *Mean Walk* (MW) model. The individual model provided a different histogram of the sample number of net time delays for the individual related pathway (step III), as shown at the bottom figure in panel B, and the averaged time delays were compared with the time delays given from neuronal spikes data (step IV).

Here, we explain how we estimated time delays in the absence of a clear stimulus onset, such as in the “Awake Task-Free condition” or the “Anesthetized condition”. This process had three steps: First, instead of evoked activity, we calculated Cross Correlations between all pairs of brain regions with de-noising of the time series, and also defined the time delays from the peak forms embedded in de-noised Cross-Correlations. Second, we identified all possible pairs of ECoG sensors located in the *Origin* and *Target* regions (Fig. 7A), and also estimated the delays for all possible pairs of ECoG sensors located in anatomically connected brain regions based on structural networks. Third, we obtained the weighted averages of the estimated the delays based on three path ensemble modes (Fig. 7B). Notice that structural networks play the essential role to estimate the propagating web, and that Cross Correlations are used for quantitative estimations of delays on directly connected individual steps. Now, let us explain more detail:

In the *first step*, we calculated Cross-Correlations after subtracting the 50 Hz components using the same notch filter as that used for evoked activities. Additionally, we subtracted cross correlations of smoothed components by the following equation:

2 $$\Vert X{C}_{corrected}\Vert =\Vert X{C}_{real}-X{C}_{smoothed}\Vert $$

*XC*_*smoothed*_ was calculated by smoothing individual waveforms using a uniform 50 ms time window. From the amplitude of the corrected cross correlations, we detected the primary peak for each pair of *Origin* and *Target* regions, within 0–30 ms. We used the time delay at the primary peak point to characterize the transmission delay of propagation on the individual step (Fig. 7A, step). This delay will be expressed as *τ*_*qp*_ in the equation 3 later.

In the *second step*, we searched all pathways connecting all combinations of ECoG sensors between the *Origin* and *Target* regions. For example, as shown Fig. 7A, we selected all ECoG sensors included in regions I and J. We call these i_1_, i_2_, i_3_, …, i_n_ and j_1_, j_2_, j_3_, …, j_m_, respectively. If a pathway from region i_2_ to region j_3_ passes through regions k and l, then we summed the time delays for the three paths: from i_1_ to k, from k to l, and from l to j_3_ (Fig. 7A, stepII).

Finally, in the *third step*, we calculated the weighted average of time delays for all detected pathways connecting regions I and J (Walk < 4). We defined the weighted average of time delays by the following equation:

3 $${\tau }_{est}=\frac{1}{{\sum }_{q=1}^{N}{{\rm{C}}}_{q}}\sum _{q=1}^{N}{{\rm{C}}}_{q}(\sum _{p=1}^{{n}_{q}}{\tau }_{qp})$$

Here, q is the index of the pathway connecting region I to region J, p is the number of Walk steps for a pathway *q*, and *n*_*q*_ is the maximum number of Walk steps. Therefore, the difference between the weights included in three Walk Ensemble models is reflected in *C*_*q*_ (Fig. 7B, stepIII). The *Shortest Path* (SP) model considers only the time delays for the shortest paths: $${{\rm{C}}}_{q}=[\begin{array}{c}1\,if\,{n}_{q}=\,{\rm{\min }}(n)\\ 0\,if\,{n}_{q} > \,{\rm{\min }}(n)\end{array}$$ (n: number of Walks). The *Mean Walk* (MW) model considers all Walks equally, so that *C*_*q*_ = 1 for any *n*. The *Decay Walk* (DW) model assigns higher weights to shorter vs longer pathways using the exponentially decaying function *C*_*q*_ = *α*^*n*^, where the decay of the exponent of *α* reflects the expectation that longer paths may be used to transmit activities less frequently than shorter paths. When *α* = 1 corresponds with the MW model, and *α* decreases toward 0, the result gradually approaches that obtained using the SP model.

Using these weighted averages (eq. 3), we obtained the representative delays given by the ECoG data under structural constraints. We compared the delays obtained from the ECoG data with the neuronal spike data reported in previous neurophysiological studies (Fig. 7B, step IV). Notice that the ECoG data had four variations related to arousal level in the Awake condition, and the light/deep Anesthetized conditions. All analyses were performed using Matlab software (The Mathworks Inc).

## Electronic supplementary material

TablesSupplementary Information
