# Author Correction: Efficient communication dynamics on macro-connectome, and the propagation speed

**DOI:** 10.1038/s41598-018-25172-7

**Published:** 2018-05-03

**Authors:** Masanori Shimono, Naomichi Hatano

**Affiliations:** 1 Graduate School of Medicine and Faculty of Medicine, Kyoto University, 53 Kawaramachi, Shogoin, Sakyo-ku, Kyoto, 606-8507 Japan; 2 Riken Brain Science Institute, 2-1 Hirosawa, Wako, Satama 351-0198 Japan; 3 Institute of Industrial Science, The University of Tokyo, Komaba 4-6-1, Meguro, Tokyo, 153-8505 Japan

Correction to: *Scientific Reports* 10.1038/s41598-018-20591-y, published online 06 February 2018

In the original version of this Article, ‘Neuroscience’ was omitted from the category list.

Additionally, in the original version of this Article, Figure 3b contained an error in the labelling of ‘Delay Walk Model’.

Finally, the original version of this Article contained an error in Figure 4a, where the tau symbol was omitted from the legend.

These errors have now been corrected in the PDF and HTML versions of the Article.

