# Supplementary material for: Efficient communication dynamics on macro-connectome, and the propagation speed
Source: Sci Rep. 2018 Feb 6;8:2510. doi: 10.1038/s41598-018-20591-y (PMC5802747; doi:10.1038/s41598-018-20591-y)
Supplement: Supplementary file 1 — TablesSupplementary Information [file 41598_2018_20591_MOESM1_ESM.doc]

**Efficient communication dynamics on macro-connectome, and the propagation speed.**

**Masanori Shimono^1,2*^, Naomichi Hatano^3^**

1. Graduate School of Medicine and Faculty of Medicine, Kyoto University, 53 Kawaramachi, Shogoin, Sakyo-ku, Kyoto 606-8507, JAPAN.
2. Riken Brain Science Institute, 2-1 Hirosawa, Wako, Satama 351-0198, JAPAN
3. Institute of Industrial Science, The University of Tokyo, Komaba 4-6-1, Meguro, Tokyo 153-8505, JAPAN

E-mail: shimono.masanori.7w@kyoto-u.ac.jp

Phone: +81-75-751-4173

**Table 1: Summary of brain region labels in four monkeys.** From left to right, the first column shows ECoG channels from the Neurochyco database. The second–fifth columns contain indexes of parcelled structural brain regions located under the ECoG sensors^41^. We compared the locations of ECoG channels and parcelled regions using Caret software^81^. Because the locations of the ECoG sensors were different among individual monkeys, the structural regions vary among them. (OrbFr: Orbital prefrontal cortex, PreM: Pre-Motor Cortex, M1: Primary Motor Cortex, SEF: Supplementary eye field, 5: Area 5, 8A: Prefrontal area 8A, 7ip: Parietal area 7ip, TA: Temporal anterior region, TAa: Area TAa, TPO: Temporal parietal occipital, V4: Visual area 4, V2: Visual area 2, V1: Primary visual area)

| **ECoG channels** | **Su** | **Georg** | **Kin2** | **Chibi** |
| --- | --- | --- | --- | --- |
| 1 | NONE | 2 | NONE | 7b |
| 2 | NONE | 7b | NONE | 7b |
| 3 | NONE | 7op | NONE | 7op |
| 4 | NONE | Toc (PA) | 9 | 7a |
| 5 | NONE | 12 | M2 (6M) | 46p |
| 6 | 9 | 6Vb | M2 (6M) | 45 |
| 7 | 9 | 6Vb | M2 (6M) | 6Val |
| 8 | M2 (6M) | 6Vb | 6Dc | 4c |
| 9 | NONE | 2 | NONE | 1 |
| 10 | NONE | 7b | NONE | 2 |
| 11 | NONE | 7b | 6Ds | 7a |
| 12 | 46p | 7op | 6DR (6D) | 7a |
| 13 | 6Ds | 7a | 6DR (6D) | V4 |
| 14 | 6Ds | 12 | 6Dc | NONE |
| 15 | 6DR (6D) | 46p | 4 | 46p |
| 16 | 6Dc | 45 | 4 | 6Ds |
| 17 | 12 | 6Val | 12 | 4 |
| 18 | NONE | 2 | NONE | 4 |
| 19 | 46v | 5v | 6Ds | 1 |
| 20 | 46p | 5v | 6Ds | 2 |
| 21 | 6Ds | 7b | 6DR (6D) | 5D |
| 22 | 6Ds | 7a | 6Dc | 7a |
| 23 | 4c | 12 | 6Dc | NONE |
| 24 | 4 | NONE | 4 | 12 |
| 25 | 2 | NONE | 4 | NONE |
| 26 | 12 | 46p | 46p | 46p |
| 27 | 46p | 46p | 46p | 6DR (6D) |
| 28 | 45 | 6Ds | 6Ds | 4 |
| 29 | 6Val | 4c | 4c | 4 |
| 30 | 4c | 2 | 4 | 1 |
| 31 | 2 | 2 | 4 | 1 |
| 32 | 2 | 5v | 2 | 5D |
| 33 | 2 | 7a | 2 | 5D |
| 34 | 5v | NONE | 7a | NONE |
| 35 | 5D | NONE | 7a | NONE |
| 36 | 6Vb | 46p | 46v | NONE |
| 37 | 6Vb | 6Ds | 46p | 6Ds |
| 38 | 6Val | 6DR (6D) | 6Ds | 6DR (6D) |
| 39 | 6Val | 6DR (6D) | 4c | 6Dc |
| 40 | 2 | 6Dc | 4 | 4 |
| 41 | 5v | 4 | 4 | 4 |
| 42 | 5v | 2 | 5v | 1 |
| 43 | 7b | 5D | 5v | 1 |
| 44 | 7a | 5D | 7a | 5D |
| 45 | 7a | NONE | 7a | NONE |
| 46 | DP | NONE | V4 | NONE |
| 47 | 6Vb | NONE | 46p | NONE |
| 48 | 6Vb | 9 | 45 | 9 |
| 49 | 6Vb | 9 | 6Val | 9 |
| 50 | 6Vb | M2 (6M) | 6Val | M2 (6M) |
| 51 | 2 | M2 (6M) | 2 | M2 (6M) |
| 52 | 5v | M2 (6M) | 2 | M2 (6M) |
| 53 | 7b | M2 (6M) | 5v | M2 (6M) |
| 54 | 7op | 6DC | 7b | 6DC |
| 55 | 7a | 4 | 7a | 4 |
| 56 | PrCO | NONE | 12 | NONE |
| 57 | PrCO | NONE | 6Vam | NONE |
| 58 | 6Vb | NONE | 6Val | 12 |
| 59 | S2 | 24b | 6Val | 24a |
| 60 | S2 | 24d | 2 | 24d |
| 61 | 7op | 24d | 2 | NONE |
| 62 | Toc (PA) | 23 | 7b | 3a |
| 63 | V4 | 10m | 7b | NONE |
| 64 | V4 | 10m | 7op | 10m |
| 65 | 6Vb | NONE | 7a | NONE |
| 66 | PrCO | 12 | 6Vb | NONE |
| 67 | PrCO | PrCO | 6Vb | NONE |
| 68 | S2 | TS (ST) | 6Vb | NONE |
| 69 | TS (ST) | TS (ST) | 6Vb | TS (ST) |
| 70 | Tpt | TAa | 7b | TS (ST) |
| 71 | V4 | TE1-3 | 7op | Taa |
| 72 | V4 | TE1-3 | Toc (PA) | TE1-3 |
| 73 | V1 | Vot | 7a | Vot |
| 74 | TS (ST) | V4 | 6Vb | V4 |
| 75 | TS (ST) | VP | 6Vb | V2v |
| 76 | TS (ST) | V1 | 6Vb | V1 |
| 77 | TAa | V1 | S2 | V1 |
| 78 | TAa | V1 | S2 | V1 |
| 79 | Vot | V1 | Tpt | V1 |
| 80 | Vot | PrCO | Tpt | NONE |
| 81 | V4 | PrCO | V4 | TS (ST) |
| 82 | V1 | TS (ST) | V4 | TS (ST) |
| 83 | V4 | TAa | V4 | TS (ST) |
| 84 | V2d | Vot | V4 | Taa |
| 85 | V2d | V4 | V4 | Vot |
| 86 | V2d | V4 | V2d | V4 |
| 87 | V1 | V2v | V4 | V2v |
| 88 | V1 | V1 | V2d | V1 |
| 89 | V1 | V1 | V2d | V1 |
| 90 | V1 | V1 | V2d | V1 |
| 91 | V1 | V1 | V1 | V1 |
| 92 | V1 | NONE | V1 | 6Vb |
| 93 | V1 | NONE | V1 | 6Vb |
| 94 | V1 | TS (ST) | V1 | 6Vb |
| 95 | TS (ST) | TS (ST) | PrCO | 2 |
| 96 | TAa | TS (ST) | PrCO | S2 |
| 97 | TAa | V4 | TS (ST) | Tpt |
| 98 | TE1-3 | V4 | TS (ST) | Tpt |
| 99 | Vot | V4 | TAa | V4 |
| 100 | V4 | V1 | V4 | V4 |
| 101 | V4 | V1 | V4 | V1 |
| 102 | VP | V1 | V1 | V1 |
| 103 | V1 | V1 | V1 | V1 |
| 104 | V1 | V1 | V1 | V1 |
| 105 | V1 | 6Vb | V1 | 6Vb |
| 106 | V1 | 6Vb | V1 | 6Vb |
| 107 | NONE | 6Vb | 12 | 6Vb |
| 108 | NONE | S2 | 6Vb | 2 |
| 109 | TS (ST) | TPOc | PrCO | TPOc |
| 110 | Taa | V1 | TS (ST) | V4 |
| 111 | TE1-3d | V1 | TS (ST) | V4 |
| 112 | TEa | V1 | TAa | V1 |
| 113 | Tea | V1 | TE1-3 | V1 |
| 114 | Vot | V1 | TE1-3 | V1 |
| 115 | V4 | V1 | Vot | V4 |
| 116 | V4 | V1 | V4 | V2d |
| 117 | VP | V1 | V4 | V1 |
| 118 | V2v | V1 | V1 | V1 |
| 119 | V1 | V1 | V1 | V2d |
| 120 | V1 | V1 | V1 | V2d |
| 121 | V1 | V1 | V1 | PIP |
| 122 | NONE | V2v | NONE | PIP |
| 123 | NONE | V1 | NONE | PIP |
| 124 | TAa | V2v | NONE | NONE |
| 125 | TE1-3d | V2d | TAa | 5D |
| 126 | TE1-3d | V2d | NONE | 31 |
| 127 | TE1-3 | V2d | NONE | 2 |
| 128 | NONE | V2d | NONE | 2 |

**Table 2: Summary of spiking neuron data:** The first column lists the abbreviated names of brain regions that could be used to assess neuronal spikes. The second–third columns are number of neuron recorded in these brain regions, and their original reference articles. The last forth column is names of parcelled structural brain regions corresponds with the brain regions used for neuronal spike recordings. LV00 is an abbreviation of the parcelled map used in Ref. 41.

| **Region name** | **Number of neurons** | **Reference** | **LV00** |
| --- | --- | --- | --- |
| V1 | 74 | 64. Schmolesky et al. (1998) | V1 |
| V2 | 61 | 64. Schmolesky et al. (1998) | V2 |
| TAa | 98 | 8. Baylis et al. (1987) | TAa |
| TPO | 547 | 8. Baylis et al. (1987) | TPOr, TPOc, TPOi |
| 7ip | 94 | 15. Chafee et al. (1998) | 7a, 7b, 7op |
| 46 | 62 | 35. Kim, Shadlen (1999) | 46p |
| Orbitofrontal | 494 | 79. Thorpe et al. (1983) | 10m |
